# Formation of the prebiotic molecule NH_2_CHO on astronomical amorphous solid water surfaces: accurate tunneling rate calculations†
†Electronic supplementary information (ESI) available: Geometric details, lists of calculated rate constants. See DOI: 10.1039/c6cp05727f



**DOI:** 10.1039/c6cp05727f

**Published:** 2016-10-06

**Authors:** Lei Song, Johannes Kästner

**Affiliations:** a Institute for Theoretical Chemistry , University of Stuttgart , Pfaffenwaldring 55 , 70569 Stuttgart , Germany . Email: kaestner@theochem.uni-stuttgart.de

## Abstract

Investigating how formamide forms in the interstellar medium is a hot topic in astrochemistry, which can contribute to our understanding of the origin of life on Earth. We have constructed a QM/MM model to simulate the hydrogenation of isocyanic acid on amorphous solid water surfaces to form formamide. The binding energy of HNCO on the ASW surface varies significantly between different binding sites, we found values between ∼0 and 100 kJ mol^–1^. The barrier for the hydrogenation reaction is almost independent of the binding energy, though. We calculated tunneling rate constants of H + HNCO → NH_2_CO at temperatures down to 103 K combining QM/MM with instanton theory. Tunneling dominates the reaction at such low temperatures. The tunneling reaction is hardly accelerated by the amorphous solid water surface compared to the gas phase for this system, even though the activation energy of the surface reaction is lower than the one of the gas-phase reaction. Both the height and width of the barrier affect the tunneling rate in practice. Strong kinetic isotope effects were observed by comparing to rate constants of D + HNCO → NHDCO. At 103 K we found a KIE of 231 on the surface and 146 in the gas phase. Furthermore, we investigated the gas-phase reaction NH_2_ + H_2_CO → NH_2_CHO + H and found it unlikely to occur at cryogenic temperatures. The data of our tunneling rate constants are expected to significantly influence astrochemical models.

## Abstract 2

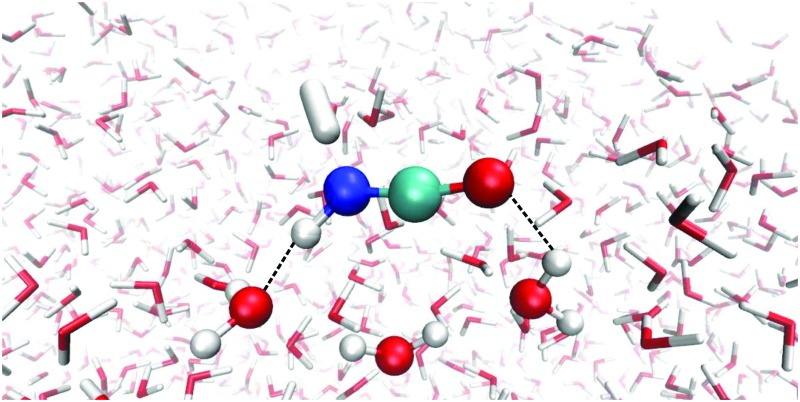
Investigating how formamide forms in the interstellar medium is a hot topic in astrochemistry, which can contribute to our understanding of the origin of life on Earth.

## 1 Introduction

Formamide (NH_2_CHO), the simplest molecule containing a peptide bond, has attracted much attention in the field of astrochemistry owing to its potential role as a prebiotic precursor in the origin of life on Earth. It was first detected in a molecular cloud in 1971 by Rubin *et al.* ^1^ Since then, formamide has been found on comets and in a variety of star-forming regions, such as in high mass young stellar objects (YSOs),^2^ outflow shock regions,^3,4^ and on the comet Hale–Bopp.^5^ Recently, López-Sepulcre *et al.* ^6^ detected NH_2_CHO in five out of ten low- and intermediate-mass pre-stellar and protostellar objects as well as isocyanic acid (HNCO) in all ten sources under study. They found a tight and almost linear correlation between NH_2_CHO and HNCO abundance, which indicates the existence of a chemical relation between those two molecules.

The formation sequence for complex organic molecules like NH_2_CHO can occur either in gas-phase or on the surface of dust grains in the interstellar medium.^7–9^ Consecutive hydrogenations of HNCO on the mantles of dust grains were proposed as a likely formation route to produce NH_2_CHO:

1 H + HNCO → NH_2_CO

2 H + NH_2_CO → NH_2_CHO

Since (2) is a radical–radical recombination reaction it is barrierless. Reaction (1) is rate-limiting and thus the focus of this study will be on it. Nguyen *et al.* ^10^ investigated (1) in the gas phase and suggested the NH_2_CO radical as the primary intermediate and NH_2_ + CO as the fragment products. However, a surface can dissipate the extra energy on the NH_2_CO radical and, thus, stabilize it. However, in recent experimental work by Noble *et al.*,^11^ the low temperature reaction of solid phase HNCO with H atoms did not produce detectable amounts of NH_2_CHO. Even though, formation of NH_2_CHO from HNCO could be possible on other surfaces, like amorphous solid water (ASW) surfaces.

A gas-phase formation route of NH_2_CHO was investigated by Barone *et al.* ^12^ using quantum chemical computations. They suggested the reaction

3 NH_2_ + H_2_CO → NH_2_CHO + H

to be barrierless and therefore a viable route for NH_2_CHO-formation in the gas phase. We will briefly address this reaction in the present work as well.

The increased concentration of active species on the surface of dust grains lends weight to the surface formation route. The mantles of dust grains are predominantly composed of H_2_O in the amorphous phase combined with other molecules such as CO, CH_4_, NH_3_, and traces of other molecules like HNCO, and NH_2_CHO. Therefore, modeling the reactions on an ASW surface is probably close to the astronomical environment.^13^ The temperature is always low on the ASW surface, where quantum tunneling is expected to play an important role in chemical reactions. In addition, quantum tunneling is also very likely to happen in the hydrogenation reactions owing to the light reactant H atoms.^14^

In this work we study reaction (1) on an ASW surface using hybrid quantum mechanics/molecular mechanics (QM/MM) calculations. Combined with instanton theory, we provide tunneling rates of this reaction in the gas phase and on the ASW surface.

## 2 Methods

### 2.1 System preparation

The ASW surface was prepared by classical molecular dynamics (MD) simulations with NAMD.^15^ The initial sample is produced by VMD version 1.9.2^16^ containing 9352 TIP3P water^17^ molecules. These were simulated in a slab of 85 Å × 85 Å and a thickness of approximately 36 Å. Periodic boundary conditions were applied along all three Cartesian axes with about 70 Å of vacuum between the slabs. This system was treated as a canonical ensemble, equilibrated at 300 K using a Langevin thermostat for 100 ps. After that, the thermostat was instantaneously quenched to 10 K and the system was left for 20 ps to produce a thermally equilibrated bulk amorphous water at low temperature. A hemisphere with a radius of 34 Å was cut out of the slab to be used in the following QM/MM calculations.

A large sample of different binding sites on the surface was generated. The HNCO molecule was placed at 113 positions on a regular 2D-grid with a step size of 2 Å covering a circular area with a radius of 12 Å. In each of the 113 points, the molecule was placed 2 Å above the surface. Water molecules with at least one atom within 6 Å were treated by QM (typically about 23 molecules), water molecules within 12 Å were optimized (typically about 161). All other molecules of the hemispheric model were frozen.

### 2.2 QM/MM method

Both geometry optimization and tunneling rate calculations were performed using a state-of-art QM/MM approach.^18,19^ In this approach, the reactants H, HNCO and their closer water surroundings were treated with density functional theory (DFT) while more distant water molecules were described by the TIP3P force field.

The hybrid QM/MM calculations^18,19^ were carried out with ChemShell,^20,21^ using an additive electrostatic embedding scheme, where the MM point charges polarize the QM electron density. We used B3LYP^22^/def2-SVPD^23^ to calculate the binding energies and binding site geometries. Different density functionals were tested and compared to coupled cluster reference values as outlined in Section 3.1. On the basis of this comparison, BHLYP-D3^24–26^/def2-TZVP^27^ was used for barriers and rate calculations. The quantum chemical program package TURBOMOLE 6.6^28^ was used for the QM part while DL_POLY^29^ built into ChemShell, was used for MM part. Force field parameters for H and HNCO (only the van der Waals parameters are used in QM/MM) were chosen in analogy to the CHARMM22 force field.^30–32^ The open-source optimizer DL_FIND^33^ was employed for geometry optimizations including the search for binding sites, the search for transition states with the dimer method^34–36^ and the determination of instanton paths using a modified Newton–Raphson approach.^37,38^

### 2.3 Instanton theory

Tunneling rates in this work were calculated using instanton theory^39–44^ in its semiclassical formulation.^37,38,43,45–51^ Instanton theory is based on statistical thermodynamics for the rate expression in which the partition function from a quantum mechanical ensemble is expressed *via* a Feynman path integral. Generally, this theory is only applicable below the crossover temperature *T*_c_:^52^
4
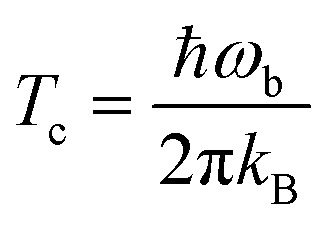
where *ω*_b_ stands for the absolute value of the classical imaginary frequency at the transition state, *k*_B_ for the Boltzmann constant and *ħ* for the reduced Planck constant. At a given temperature below *T*_c_, the instanton itself is the tunneling path with the highest statistical weight, which can be located using standard approaches for finding transition states.^37,38^ Integrating along this path and combining it with the partition function of reactant state, we can calculate instanton rate constants which consider quantum tunneling effects. Due to its semi-classical nature, instanton theory can offer a reasonable ratio of accuracy *versus* computational cost, appropriate for our reactions with organic molecules on the ASW surface. Instanton theory is meanwhile frequently used to calculate reaction rates in different areas of chemistry.^14,37,53–74^

The Feynman paths were discretized to 40 images at *T* ≥ 135 K and 78 images at lower temperature. Convergence was checked rigorously, *e.g.* at 100 K doubling the number of images changed the rate constant by only 2%.

In order to make our calculated rate constants accessible to astromodellers, we fitted them to a rate expression proposed previously:^75^
5
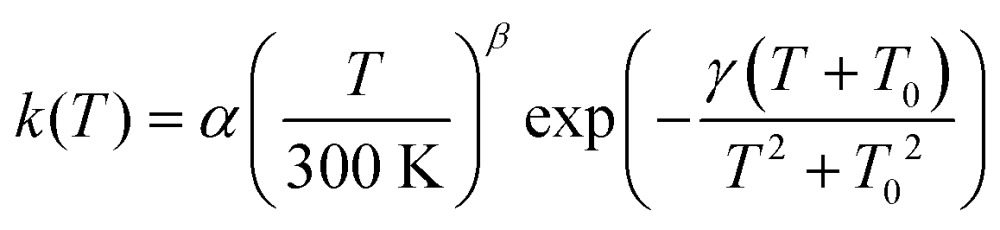
in which *α*, *γ* and *T*_0_ were used as fitting parameters and *β* was set to one. The pre-exponential factor *α* has the same unit as the rate constant and can be interpreted as an attempt frequency. The parameter *γ* is related to the barrier height and *T*_0_ is a temperature, which relates to the onset of strong tunneling. Any physical meaning of these fitting parameters should not be over interpreted, though. Instanton rate constants were used for the fit below *T*_c_, rate constants calculated by transition state theory with vibrations treated by quantum harmonic oscillators and a symmetric Eckart barrier for tunnel corrections were used to fit above *T*_c_. Eqn (5) describes classical thermal reactions as well as tunneling rates with a single expression. For *T*_0_ → 0 it turns into the standard Arrhenius equation which is used in many astrochemical models.

## 3 Results

### 3.1 Benchmark calculations

**Fig. 1:**
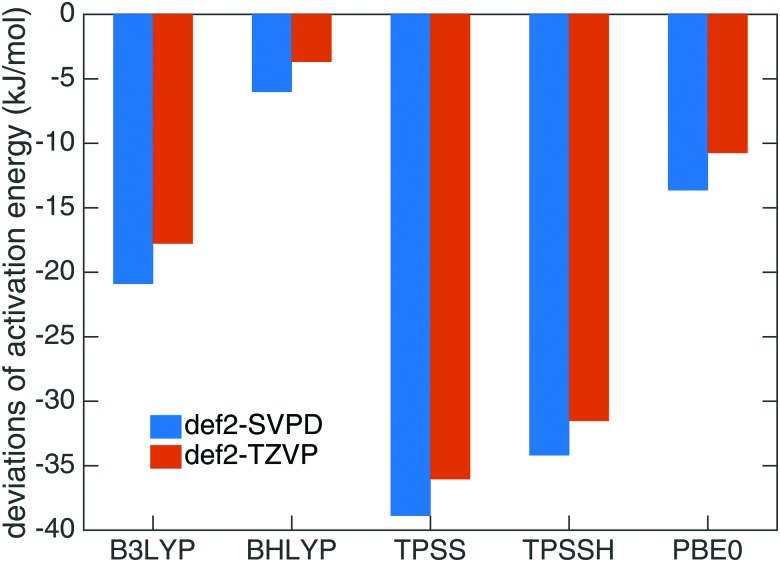
Deviations of activation energies of reaction (1) at different DFT levels with D3 dispersion correction from the results at UCCSD(T)-F12/cc-pVTZ-F12 level.

Benchmark calculations were performed to choose a proper DFT functional for the transition state search and tunnel rate calculations. We calculated the activation energy *E*_a_ for reaction (1) in the gas phase based on B3LYP-D3^22,26^/def2-TZVPD^23^ optimized geometries using UCCSD(T)-F12^76,77^/cc-pVTZ-F12^78^ on a RHF reference in MOLPRO 2012.^79^ The resulting *E*_a_ of 32.7 kJ mol^–1^ was used as a reference and compared to the data from B3LYP, BHLYP, TPSS, TPSSH and PBE0 functionals with the def2-SVPD^23^ and def2-TZVP^27^ basis sets. All DFT calculations include D3 dispersion corrections.^26^ The results are compared in Fig. 1. The smallest deviation was found for the BHLYP-D3^24–26^/def2-TZVP^27^ theory level which we selected as the proper quantum mechanical level for QM molecules.

### 3.2 HNCO binding sites and binding energies

**Fig. 2:**
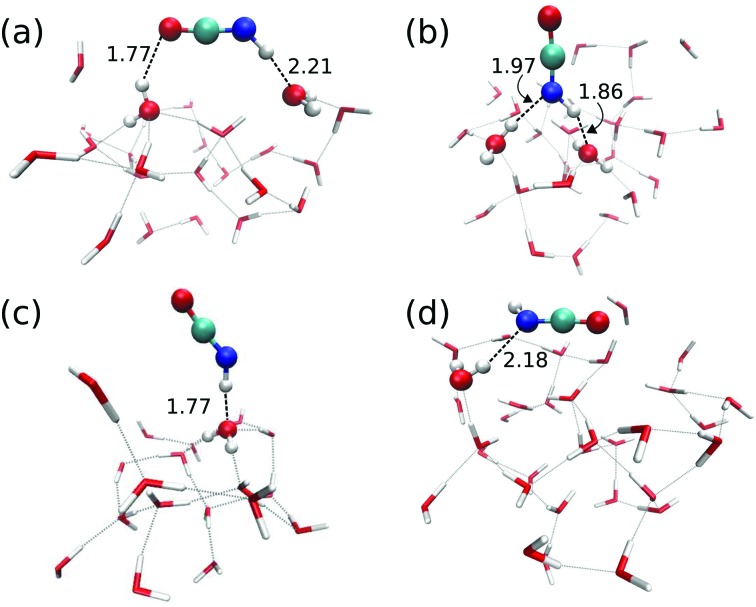
Four different HNCO binding modes on the amorphous solid water surface. Only QM molecules are shown, HNCO and all water molecules H-bonded to it are shown as ball-and-stick. Bond distances are given in Å.

 Reaction (1) originates from HNCO bound to the ASW surface. We investigated different binding modes and their respective binding energies in our QM/MM setup using the B3LYP^22^/def2-SVPD^23^ level for the QM calculations. Geometry optimization was performed starting from 113 initial structures. Among those, 90 jobs finished successfully and provided four types of HNCO binding modes on the ASW surface as shown in Fig. 2. Panel (a) illustrates the major adsorption mode to which 48 out of the 90 cases belonged. In this case the H and O ends of the HNCO molecule act as H-bond donor and acceptor connecting to O and H atoms in the water ice, respectively. The N atom can also act as a H-bond acceptor while the H atom of the HNCO molecule still serves as a H-bond donor to connect to an O atom from the water. This case is depicted in panel (b) of Fig. 2 and accounts for 34 of 90 cases. The remaining 8 cases resulted in binding modes where either the N atom or H atom in the HNCO molecule connects to H or O of the surface, as shown in panels (c) and (d).

**Fig. 3:**
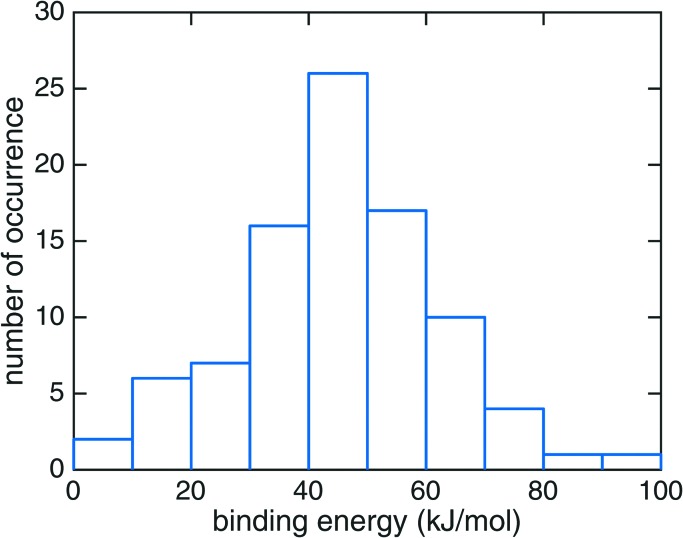
The distribution of HNCO binding energies on the amorphous solid water surface at the B3LYP^22^/def2-SVPD^23^ level of theory.

The binding energy of HNCO on the ASW surface was the energy required to disassemble the adsorbed HNCO from the surface into the gas phase. The minima of the ASW surface with and without HNCO in each of the 90 cases were calculated using the same QM, active and frozen water regions. Fig. 3 presents the distribution of binding energies from the 90 cases. It is obvious that the binding energy is very broadly distributed from 0 to about 100 kJ mol^–1^ with the largest fraction between 40 and 50 kJ mol^–1^. The tighter bound sites are expected to be occupied preferentially, which leads to a surface-coverage dependent binding energy. No clear correlation can be found between the binding modes distinguished in Fig. 2 and the binding energies. The rough surface of ASW leads to the significant spread of binding energies, which likely is of relevance for astrochemical modeling of adsorption and desorption processes. The binding energies are given in Fig. 3 without considering the vibrational zero point energy (ZPE). We calculated the ZPE for the four representative modes shown in Fig. 2. They reduce the binding energy by 8.0, 5.4, 2.3, and 7.7 kJ mol^–1^ for the modes a, b, c, and d, respectively. Thus, the influence of the ZPE on binding is small.

### 3.3 Transition states

**Fig. 4:**
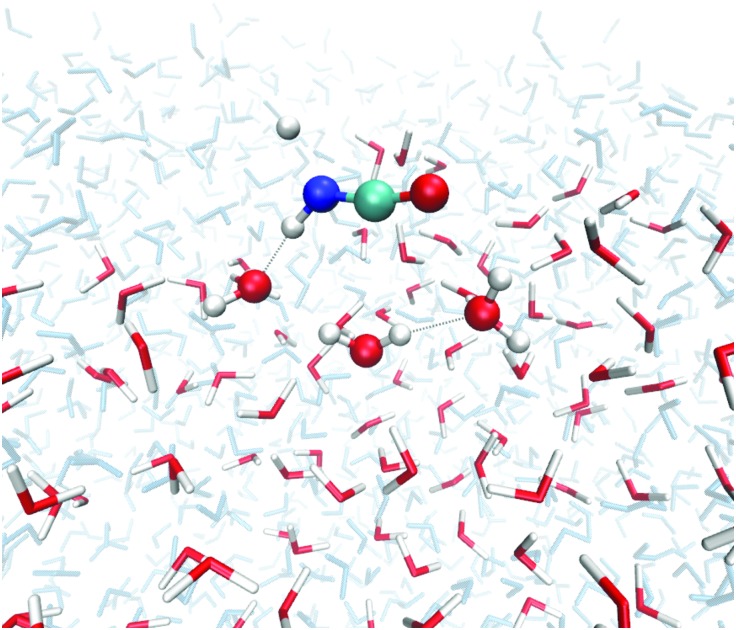
Optimized geometry of TS1 of reaction (1) on the ASW surface. In the TS search the QM region was restricted to the molecules shown as ball-and-stick models. All red/white water molecules were active, the blue/gray ones frozen.

We investigated transition states for four different binding geometries with rather different binding energies. The resulting data are given in Table 1. The transition structures are labeled TS1 to TS4. Their binding energies differ between 27.9 and 80.3 kJ mol^–1^. The attack by a hydrogen atom at the N-site of HNCO requires the latter to be accessible. Thus, binding modes (a) and (c) of the ones depicted in Fig. 2 are most promising. TS1, TS3, and TS4 correspond to binding mode (a) while TS2 corresponds to binding mode (c). For the transition state search and the following tunneling rate calculations, we restricted the QM region to H + HNCO plus just three water molecules (5 for TS2, 4 for TS4), see Fig. 4. While the same set of atoms (12 Å) was optimized as in the investigations of the binding sites, the Hessian calculations were restricted to the QM region.

**Table 1:** Comparison of transition states in gas and on the amorphous solid water surface. The energies are given in kJ mol^–1^, frequencies in cm^–1^, temperatures in K and bond distances in Å

	Gas TS	ASW			
		TS1	TS2	TS3	TS4
HNCO binding energy		48.1	27.9	80.3	52.1
N–H bond distance	1.542	1.546	1.532	1.546	1.547
*ω*_b_	1339*i*	1240*i*	1271*i*	1268*i*	1262*i*
*E*_a_ (ER mechanism)	30.6	26.7	29.9	28.4	27.9
*E*_a_ incl. ZPE	36.2	31.8	36.5	32.7	32.7
*T*_c_	307	284	291	290	289

All data in Table 1 refer to a reactant state with HNCO adsorbed on the surface and H in the gas phase, *i.e.* to an Eley–Rideal-type (ER) surface reaction mechanism. Compared with the transition state in the gas phase, the ones on the ASW surface have slightly lower activation energies *E*_a_. Without ZPE the four surface-bound activation energies are 3.9 to 0.7 kJ mol^–1^ lower than the gas-phase *E*_a_, including ZPE they are between 4.4 kJ mol^–1^ lower and 0.3 kJ mol^–1^ higher. Note that despite the large spread in binding energies of the different adsorption sites, the associated activation energies are very similar. This indicates similar rate constants, which will be discussed in the following section. The N–H bond distances of the transition states on the surface are generally slightly longer than in the gas phase, see Table 1, indicating an earlier transition state on the surface.

The transition states TS1, TS3, and TS4 describe a movement of the hydrogen atom coming from the gas phase above the surface. By contrast in TS2, which originates from a structure like the one in Fig. 2(c), the hydrogen atom approaches the nitrogen site from closer to the surface, see also Fig. S1 of the ESI.† In this case, a well-defined pre-reactive minimum with H loosely bound to the surface was found. This corresponds to a possible reactant site for a Langmuir–Hinshelwood (LH) mechanism. The barrier with respect to the LH reactant state is 34.6 kJ mol^–1^ (37.9 kJ mol^–1^ with ZPE).

### 3.4 Tunneling rate constants

**Fig. 5:**
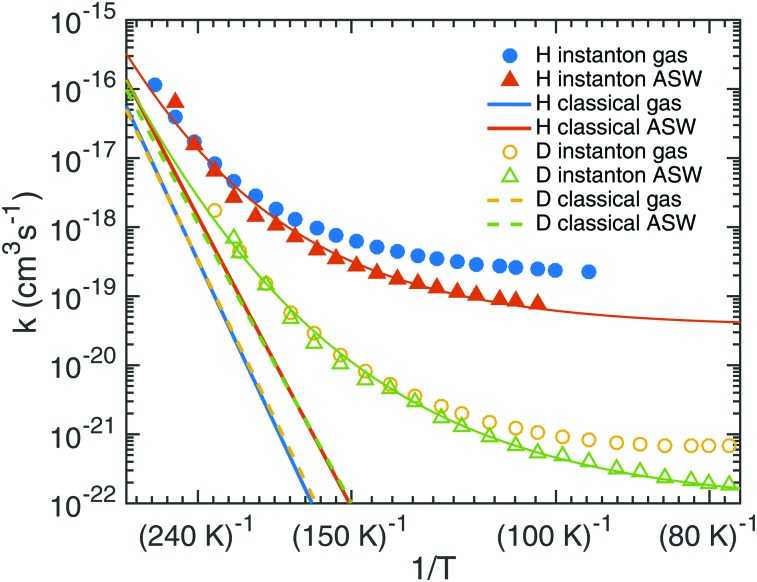
Instanton and classical rate constants for the reactions of H + HNCO → NH_2_CO and D + HNCO → NHDCO in gas and the ER process on the ASW surface. The thin lines represent fits using eqn (5).

**Fig. 6:**
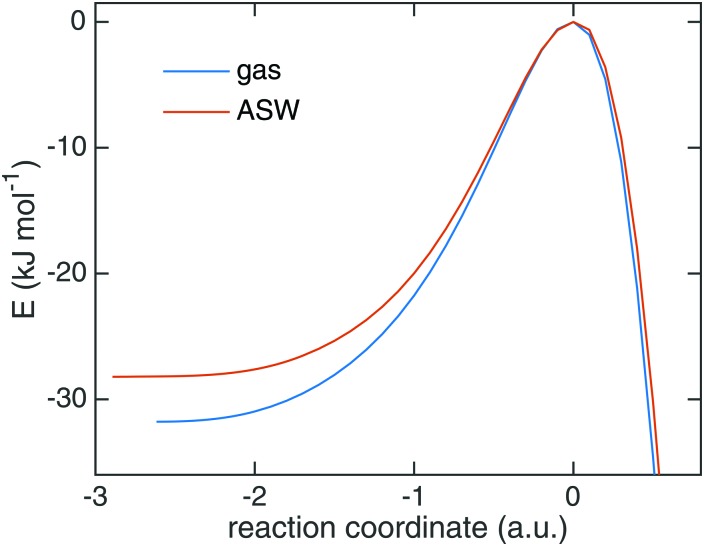
The minimum energy path of the reaction of H + HNCO → NH_2_CO in the gas phase and on the amorphous solid water surface.

Starting from TS1 we calculated rate constants for reaction (1) following an ER mechanism on the ASW surface and compared them to the gas phase reaction treated at the same QM level of theory. The results are shown in Fig. 5. The red solid triangles correspond to the rate constants on the ASW surface, the blue solid circles to the ones of the corresponding gas-phase reaction. Instanton rate constant calculations are restricted to temperatures below *T*_c_. At high temperature the surface-bound reaction is slightly faster than the gas-phase reaction; at low temperature the case is reversed and the gas-phase reaction becomes more efficient. Thus, there is no significant catalytic effect of the surface. However, the surface of course still has the effect of dissipating the excess energy of the reaction and increasing the local concentration of the reactants. Despite the lower barrier, the tunneling rate constant for the ASW-bound reaction is lower than the gas phase reaction at low temperature. This demonstrates again that besides the barrier height, the barrier width is important for the tunneling efficiency.^71^ The barrier shapes along the intrinsic reaction coordinates (IRC) are compared in Fig. 6, which clearly shows that the ASW-barrier is lower but broader than the gas-phase barrier which leads to the lower tunneling rate at low temperature.

Our data allow the comparison between a structural model which contains the surface explicitly and a gas-phase model for the surface reaction. As discussed above, the barrier changes only very slightly due to the influence of the surface and quite independently of the binding site. The resulting rate constants are very similar. The surface, however, restricts the rotational motion of the reactant and the transition state. The change in the rotational partition function is included in the rate constants depicted in Fig. 5. One can model a surface by considering only the atoms HNCO + H explicitly but restricting the rotational motion, *i.e.* ignoring the change in the rotational partition function between HNCO and the transition state. This corresponds to the rotational restriction of both HNCO and the transition state on the surface. With such an approach, the rate constants obtained from a gas-phase model are even more similar to those obtained from the surface model, *e.g.*, at 103 K we find a rate constant on the surface of 7.8 × 10^–20^ cm^3^ s^–1^, of 8.0 × 10^–20^ cm^3^ s^–1^ for the gas phase model with restricted rotation and of 2.4 × 10^–19^ cm^3^ s^–1^ for the gas phase model with full rotation. For the reaction under study a gas-phase model with restricted rotation results in sufficiently accurate surface rate constants.

Rate constants were fitted to eqn (5) to facilitate the use of our results in astrochemical models. The parameters are given in Table 2, the resulting curves are shown in Fig. 5 as thin red and green lines. They match the calculated rate constants reasonably well. We recommend using the fit in a temperature range close to the range that was used to produce it, *i.e.* 1000 K to ∼90 K for H + HNCO and 1000 K to ∼60 K for D + HNCO.

**Table 2:** Parameters for rate constants described of the reaction H/D + HNCO by eqn (5)

Parameter	H	D
*α* (cm^3^ s^–1^)	7.22 × 10^–12^	4.13 × 10^–12^
*β*	1	1
*γ* (K)	2856	2887
*T*_0_ (K)	195.8	153.4

The red and blue straight lines in Fig. 5 correspond to the rate constants neglecting tunneling (but including quantized vibrations and, thus, the ZPE). Due to the smaller barrier, without tunneling the surface-bound reaction is always faster than the gas-phase reaction. Tunneling accelerates the reaction by many orders of magnitude at low temperature. Values for the rate constants with and without tunneling are given in Tables S2 and S3 of the ESI.† For example at 103 K, tunneling accelerates the gas-phase by a factor of 2 × 10^10^ and the surface reaction by a factor of 10^8^. These values increase steeply with decreasing temperature.

**Fig. 7:**
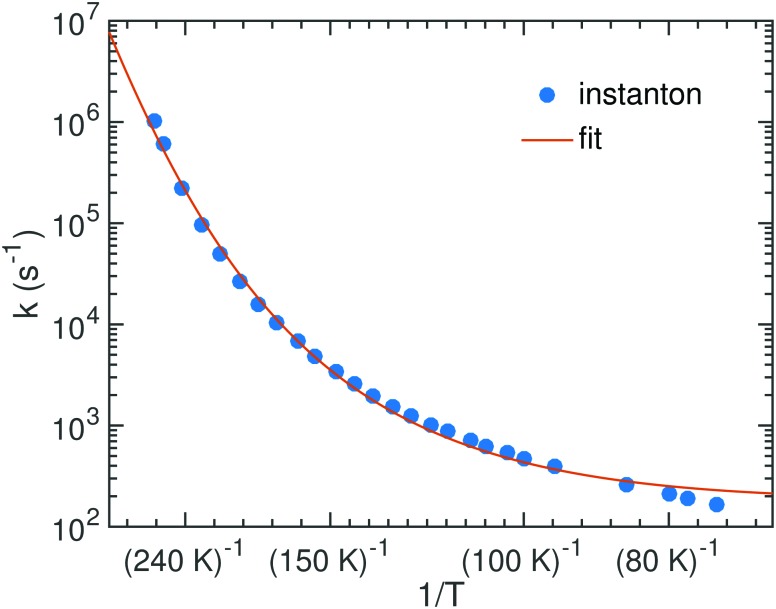
Instanton rate constants for the LH process of reaction (1) on the ASW surface.

The bimolecular rate constants reported above relate to an ER mechanism. At low temperature a LH mechanism is more likely. In that case we can assume HNCO to be stationary on the surface while the H atom diffuses with the hopping rate constant *k*_hop_ until it meets a HNCO site. Then it can either react or diffuse away again. The probability for reaction is *k*_react_/(*k*_react_ + *k*_hop_) where *k*_react_ is a unimolecular rate constant which we can calculate. It corresponds to the process of an encounter complex of H with HNCO reacting to NH_2_CO. Since H is bound very weakly on the surface, we were able to optimize such an encounter complex only for TS2. Its energy is 4.7 kJ mol^–1^ (1.4 kJ mol^–1^ with ZPE) below that of the separated reactants. The resulting rate constants are shown in Fig. 7. We fitted the parameters of eqn (5), which resulted in *α* = 3.56 × 10^10^ s^–1^, *γ* = 2503 K and *T*_0_ = 172.9 K. The parameter *β* was kept to 1 just like in the other fits.

### 3.5 Kinetic isotope effects

In addition, we investigated the kinetic isotope effect (KIE) for reaction (1). For D + HNCO → NHDCO, the crossover temperature is reduced from 284 K to 218 K on the ASW surface and from 307 K to 235 K in the gas phase. In Fig. 5 instanton rate constants for the reactions with deuterium in the gas-phase are shown by yellow circles and on ASW by green triangles. Similar trends are visible as for the addition of protium to HNCO, but the rate constants are much smaller. As frequently observed for tunneling reactions, the KIE increases with decreasing temperature. At 103 K the KIE for the gas-phase reaction is 231, on the ASW surface it is 146. Even stronger KIEs can be expected at lower temperature. The KIEs without tunneling are much smaller as can be seen from Fig. 5, which indicates that the KIE is mostly caused by tunneling rather than by the difference in the ZPE.

### 3.6 Alternative gas-phase reaction

To elucidate a possible role of reaction (3) for the formation of NH_2_CHO, we calculated the barrier for the initial reaction channel, the approach of NH_2_ to formaldehyde. We optimized the reactants and the transition state on the M06-2X^80^/def2-TZVP^27^ level using NWCHEM 6.6^81^ and calculated single-point energies and vibrational frequencies on the UCCSD(T)-F12^76,77^/cc-pVTZ-F12^78^ level. The coordinates of the transition structure are given in the ESI.† In agreement with previous work,^12^ we found an almost submerged barrier on the potential energy surface, +2.7 kJ mol^–1^ compared to the separated reactants. Including the ZPE, however, resulted in a significant barrier of 17.8 kJ mol^–1^. The crossover temperature is 88.0 K. Thus, tunneling only plays a minor role above that temperature. The corresponding rate constant for reaction (3) at 100 K is *k* = 1.1 × 10^–22^ cm^3^ s^–1^ if tunneling is neglected and quite a similar value of *k* = 5.3 × 10^–22^ cm^3^ s^–1^ if tunneling is approximated *via* a symmetric Eckart barrier. Note that above the crossover temperature, instanton theory is not applicable. These rate constants can only serve as an upper limit to the full rate constant of reaction (3) since they only cover the entrance channel. The full reaction contains additional submerged barriers^12^ which might lower the rate even further. Nevertheless, even these upper bounds are significantly smaller than the rate constant of *k* = 2.4 × 10^–19^ cm^3^ s^–1^ for reaction (1) at the same temperature. Thus, we conclude that the gas-phase reaction (3) is expected not to play a significant role in the formation of NH_2_CHO.

## 4 Conclusions

We investigated binding of HNCO to an ASW surface and subsequent hydrogenation. Different binding sites with a significant spread of binding energies were found. The activation barrier for the hydrogenation reaction turned out to be rather independent of the binding energy. We calculated the reaction rate constants for H + HNCO → NH_2_CO in the gas phase at temperatures of 289 K down to 95 K and on the ASW surface down to 103 K by combining the QM/MM method with instanton theory. Although the activation barrier for the surface reaction is 3.9 kJ mol^–1^ (4.4 kJ mol^–1^ including ZPE) lower than in the gas-phase, the ASW surface does not efficiently accelerate this reaction, but hinders it at temperatures below 240 K. It demonstrates that the width but not the height of the barrier dominantly affects the tunneling rate for this system. In addition, the deuterated reaction of D + HNCO → NHDCO has been investigated both in the gas-phase and on the ASW surface. According to the instanton calculations, the KIEs are 231 and 146 for the gas phase reaction and the surface reaction at 103 K, respectively and expected to be at least similarly strong at even lower temperature. The strong tunnel effect raises the rate constants to values which enable hydrogenation of HNCO on the surface of interstellar dust grains, making this a possible route for the formation of the pre-biotic molecule formamide. By contrast, the gas-phase route *via*reaction (3) seems inaccessible at low temperature.
