# Supplementary material for: Formation of the prebiotic molecule NH2CHO on astronomical amorphous solid water surfaces: accurate tunneling rate calculations
Source: Phys Chem Chem Phys. 2016 Oct 6;18(42):29278–85. doi: 10.1039/c6cp05727f (PMC5317214; doi:10.1039/c6cp05727f)
Supplement: Supplementary file 1 [file CP-018-C6CP05727F-s001.pdf]

Electronic Supplementary Material (ESI) for Physical Chemistry Chemical Physics.
This journal is © the Owner Societies 2016

Supplementary information to:
Formation of the prebiotic molecule NH_2CHO on astronomical amorphous solid water surfaces: accurate tunneling rate calculations

Lei Song, Johannes Kästner

Cartesian coordinates of stationary points:

HNCO

```
N   -0.8975531  -0.1593249   0.0000000
C    0.2988332  -0.2178045   0.0000000
O    1.4340317  -0.4021137   0.0000000
H   -1.4556322   0.6654845   0.0000000
```

TS of $H + HNCO \longrightarrow NH_2CO$

```
N   -2.3102196  -0.3766682  -0.0000004
C   -1.0930308  -0.4013962   0.0000008
O    0.0149439  -0.7212172  -0.0000004
H   -2.8822644   0.4379437   0.0000088
H   -3.2446203  -1.6032927  -0.0000053
```

TS of $NH_2 + H_2CO \longrightarrow NH_2CH_2O$

```
N    0.465815    0.077152    0.623183
H    0.537642    1.087717    0.471744
H   -0.450786   -0.170730    0.239061
C    0.112568   -0.001936    2.600317
O   -0.821334    0.794605    2.582987
H    1.127538    0.311995    2.881437
H   -0.062542   -1.087154    2.607181
```

Table 1 Benchmark calculations for the reaction of H+HNCO $\rightarrow$ NH_2CO. (unit of energy: kJ/mol)

Theory	Basis-set	Activation Energy	Deviation
CCSD(T)-F12	VTZ-F12	32.7	
B3LYP	def2-SVPD	11.8	-20.9
	def2-TZVP	15.0	-17.8
BHLYP	def2-SVPD	26.7	-6.0
	def2-TZVP	29.0	-3.7
TPSS	def2-SVPD	-6.2	-38.9
	def2-TZVP	-3.3	-36.0
TPSSH	def2-SVPD	-1.5	-34.2
	def2-TZVP	1.2	-31.5
PBE0	def2-SVPD	19.1	-13.6
	def2-TZVP	22.0	-10.7

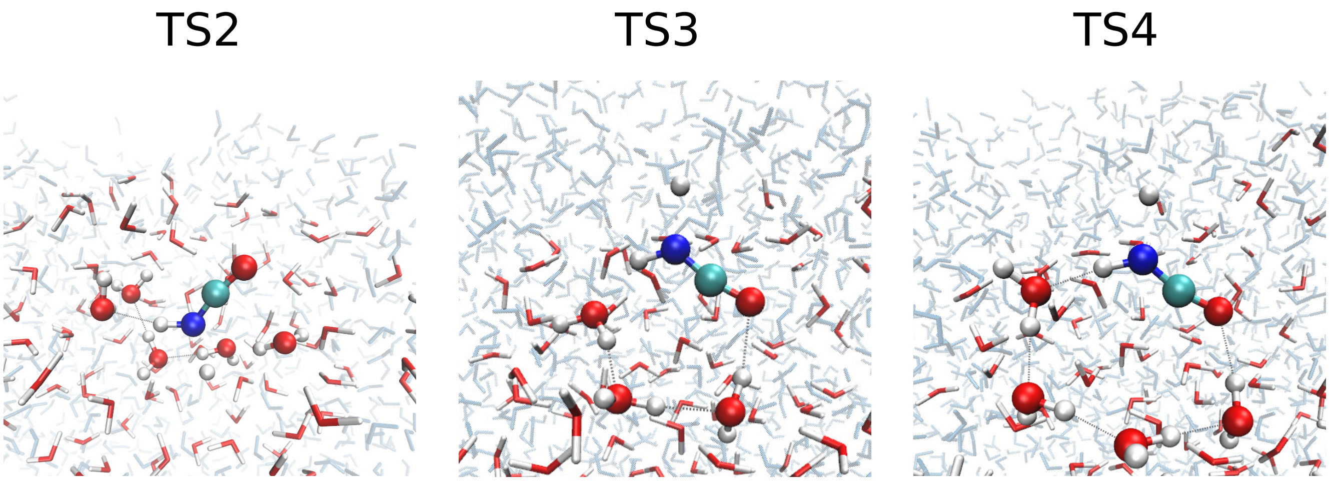

Fig. 1 Optimized structures of three transition states labeled as TS2, TS3 and TS4 at BHLPY/def2-TZVP theory level with D3 dispersion correction. For geometry optimization, the ball-and-stick molecules were in the QM region. The grey and red sticks stand for the active MM water molecules while the blue sticks represent the frozen MM water molecules. For Hessian analysis, the ball-and-stick molecules are still in the QM region, but all other water molecules are in the frozen MM region.

Table 2 Classical rate constants Log(k) (k in cm^3s^{-1}) for the reactions of $H + HNCO \longrightarrow NH_2CO$ and $D + HNCO \longrightarrow NHDCO$ in gas and on the amorphous solid water surface.

T(K)	H gas	H ASW	T(K)	D gas	D ASW
378.95	−15.55	−15.23	350.00	−16.03	−15.77
360.00	−15.82	−15.48	331.58	−16.33	−16.03
342.86	−16.09	−15.72	315.00	−16.63	−16.30
327.27	−16.36	−15.96	300.00	−16.93	−16.56
313.04	−16.63	−16.19	286.36	−17.22	−16.83
300.00	−16.90	−16.43	273.91	−17.52	−17.09
288.00	−17.16	−16.67	262.50	−17.81	−17.35
276.92	−17.43	−16.91	252.00	−18.11	−17.62
266.67	−17.69	−17.14	242.31	−18.4	−17.88
257.14	−17.96	−17.38	233.33	−18.69	−18.14
248.28	−18.22	−17.62	225.00	−18.99	−18.40
240.00	−18.49	−17.85	217.24	−19.28	−18.66
232.26	−18.75	−18.09	210.00	−19.57	−18.91
225.00	−19.01	−18.32	203.23	−19.86	−19.17
218.18	−19.28	−18.56	196.88	−20.15	−19.43
211.76	−19.54	−18.79	190.91	−20.45	−19.69
205.71	−19.80	−19.02	185.29	−20.74	−19.95
200.00	−20.07	−19.26	180.00	−21.03	−20.20
194.59	−20.33	−19.49	175.00	−21.32	−20.46
189.47	−20.59	−19.72	170.27	−21.61	−20.72
184.62	−20.85	−19.96	165.79	−21.90	−20.97
180.00	−21.12	−20.19	161.54	−22.18	−21.23
175.61	−21.38	−20.42	157.50	−22.47	−21.48
171.43	−21.64	−20.66	153.66	−22.76	−21.74
167.44	−21.90	−20.89	150.00	−23.05	−22.00
163.64	−22.16	−21.12	145.00	−23.47	−22.37
160.00	−22.42	−21.35	137.00	−24.20	−23.01
156.52	−22.69	−21.58	130.00	−24.92	−23.64
153.19	−22.95	−21.82	123.00	−25.71	−24.34
150.00	−23.21	−22.05	118.00	−26.33	−24.89
148.00	−23.38	−22.20	112.00	−27.16	−25.62
141.00	−24.01	−22.76	107.00	−27.91	−26.29
135.00	−24.60	−23.28	103.00	−28.57	−26.87
129.00	−25.25	−23.86	99.00	−29.28	−27.49
124.00	−25.84	−24.38	95.00	−30.05	−28.17
119.00	−26.47	−24.94	91.00	−30.89	−28.91
115.00	−27.02	−25.43	88.00	−31.57	−29.51
110.00	−27.76	−26.09	85.00	−32.29	−30.14
107.00	−28.24	−26.51	82.00	−33.07	−30.83
103.00	−28.92	−27.11	80.00	−33.63	−31.32
100.00	−29.47	−27.60	78.00	−34.21	−31.83
95.00	−30.46	−28.47			

Table 3 Instanton rate constants Log(k) (k in cm^3s^{-1}) for the reactions of $H + HNCO \longrightarrow NH_2CO$ and $D + HNCO \longrightarrow NHDCO$ in gas and on the amorphous solid water surface.

T(K)	H gas	H ASW	T(K)	D gas	D ASW
289.00	−15.94		225.00	−17.77	
264.00	−16.40	−16.19	210.00		−18.17
243.00	−16.77	−16.82	206.00	−18.34	−18.37
225.00	−17.08	−17.18	190.00	−18.82	−18.83
210.00	−17.33	−17.58	176.00	−19.23	−19.32
196.00	−17.56	−17.85	165.00	−19.54	−19.67
184.00	−17.74	−17.97	154.00	−19.85	−19.98
174.00	−17.88	−18.13	145.00	−20.08	−20.21
164.00	−18.02	−18.33	137.00	−20.28	−20.33
156.00	−18.12	−18.45	130.00	−20.45	−20.52
148.00	−18.21	−18.56	123.00	−20.60	−20.76
141.00	−18.29	−18.67	118.00	−20.71	−20.90
135.00	−18.34	−18.75	112.00	−20.82	−21.04
129.00	−18.42	−18.82	107.00	−20.91	−21.17
124.00	−18.46	−18.88	103.00	−20.97	−21.27
119.00	−18.50	−18.93	99.00	−21.03	−21.32
115.00	−18.53	−18.99	95.00	−21.08	−21.40
110.00	−18.57	−19.04	91.00	−21.12	−21.50
107.00	−18.58	−19.08	88.00	−21.14	−21.55
103.00	−18.61	−19.11	85.00	−21.16	−21.64
100.00	−18.62		82.00	−21.17	−21.67
95.00	−18.65		80.00	−21.17	−21.72
			78.00	−21.17	−21.74

Table 4 Unimolecular rate constants Log(k) (k in s^{-1}) for the reaction of $H + HNCO \longrightarrow NH_2CO$ on the amorphous solid water surface.

T(K)	Log(k)	T(K)	Log(k)	T(K)	Log(k)
275.00	6.02	156.00	3.68	107.00	2.80
264.00	5.79	148.00	3.54	103.00	2.73
243.00	5.34	141.00	3.41	100.00	2.67
225.00	4.98	135.00	3.30	95.00	2.59
210.00	4.70	129.00	3.18	85.00	2.41
196.00	4.43	124.00	3.09	80.00	2.32
184.00	4.20	119.00	3.01	78.00	2.28
174.00	4.01	115.00	2.94	75.00	2.23
164.00	3.83	110.00	2.85		
